# A Case of Hydrophobia

**Published:** 1787

**Authors:** David Dundas

**Affiliations:** Surgeon at Richmond, in Surry


X. *A Caſe of Hydrophobia. By Mr.* David Dundas, *Surgeon at Richmond, in Surry. Communicated in a Letter to* John Grieve, *M. D. Member of the Royal College of Phyſicians of London, and by him to Dr.* Simmons.

HENRY Rider, a ſtout, healthy man, aged about forty years, was bit in the wriſt, in the month of Auguſt, 1785, by a little fox dog belonging to himſelf, which he immediately ſhot, it having bit a pig, and ſhewn an inclination to bite all who came near it. He did not apply to me till ſome hours after the bite; and as ſo much time had elapſed, and the wound was inconſiderable, and there was was no certain proof of the madnefs of the dog, I dreffed it only with fome common cerate. I advifed him, however, to take the Ormfkirk powder, which he did the next morning; and he alfo applied the powder, mixed with vinegar, to the wound, according to the printed directions. As he expreffed an inclination to be dipped in the fea, I encouraged him in it, and he proceeded to Gravefend, where he underwent the ufual operation of immerfion.

From that time he remained perfectly well, and continued to follow his occupation of keeping an alehoufe in this neighbourhood till about the end of December, 1786, at which time, finding his bufinefs unfuccefsful, and being apprehenfive of an arreft, he quitted his fituation, and went to London in order to conceal himfelf from his creditors till his affairs could be fettled.

On Friday, the 23d of February, 1787, he dined with the fervants of a gentleman in London, about feven o'clock in the evening: he ate fome boiled tripe heartily; but upon attempting to drink fome porter, he was furprifed he could not fwallow it, and imagined a piece of the tripe had ftuck in his throat; he tried again to drink, and was almoft ftrangled in the atempt; tempt; but was able to chew fome bread, and to fwallow it eafily, though he ftill experienced the fame difficulty as before with any liquid. He went home very uneafy; had fome flight rigours, and felt fuch an anxiety and oppreffion, that he could not go to bed. In the morning he ate fome buttered toaft, but could not fwallow any tea. He now applied to an apothecary in London, who bled him, and gave him fome purging pills; but as he hourly became worfe, he fet out for Richmond, and called on me about nine o'clock on Saturday evening, February the 24th.

He had been apprehenfive that the difficulty of drinking might be the confequence of the bite, as I learned from a converfation he had had with his wife, but he ftill flattered himfelf it might proceed from fome of the tripe fticking in his throat, and he told me he had come to me in hopes I could pufh it down; but upon my afking him if he had ever felt any uneafinefs in the wrift which was bit, he told me he perceived what my opinion was, and was ready to fubmit to his fate.

He appeared very much agitated and oppreffed; complaining moft of a weight at his ftomach, and of an anxiety which he could not defcribe; describe: his pulse was very quick and small; his eyes looked wild: he complained of a little head-ach, and had a great dryness in his mouth, but dreaded the sight of drink. Upon shewing him a glass of water, he appeared much agitated, but said he was determined to drink it; with difficulty he got it to his mouth, but not a drop of it was taken in, and his whole body was violently convulsed. Some of the water had been spilt upon the table, and his hand by chance touching it, produced nearly the same effects.

He told me he had observed an itching in his wrist for some days, but it did not appear at all discoloured or swelled.

As he had already had the hydrophobia upwards of twenty-seven hours, I did not suppose any thing could relieve him. A large blister, however, was applied to his back; an ounce of mercurial ointment was directed to be rubbed into his arms and legs every two hours; and a clyster, containing an ounce of the tinctura fœtida, and fifty drops of laudanum, was given every four hours. Dr. Prendergast, who saw him along with me, wished him to take the turpeth mineral; but it was impossible for him now to swallow it.

On Sunday morning, the 25th, he was very ſenſible, but had not been in bed all night: he retired to a corner of the room; looked very wild; his pulſe was very quick; and he frequently made a noiſe very like the barking of a dog. He told the people who were with him not to be afraid, as he ſhould not hurt them; but he did not like to be ſpoke to; and when any ſtranger came into the room, it agitated him much.

About the middle of the day he attempted to bite and ſtrike the people who were attending him, and became ſo much convulſed, that it was neceſſary to put a ſtrait waiſtcoat on him. After a violent ſtruggle, he became much more ſerene. The mercurial friction was continued without intermiſſion, but without any ſenſible effect; and from this period his ſituation was truly deplorable. He imagined he was to be ſmothered betwixt two featherbeds, and every time I came to ſee him he apprehended it was to give the fatal order.

No perſuaſion could remove this unhappy idea from his mind; and he evidently ſuppreſſed his complaints, in order to conceal, as he ſuppoſed, from me the neceſſity of my proceeding to the laſt extremity. This diſtreſs of mind mind feemed much greater than his bodily pain; and had it not been for the former, I fhould have fuppofed him to fuffer much lefs than perfons do in many other difeafes.

From the evening till betwixt three and four o'clock on Monday morning he continued perfectly fenfible, gave orders about his funeral, and having taken leave of his wife, was feized with a convulfion, which, in a few minutes, put a period to his fufferings.

I examined the body the next day; but upon opening the head, no appearance of difeafe could there be difcovered. The veffels of the brain did not feem more turgid with blood than ufual; neither was any inflammation or fwelling difcoverable about the pharynx or neighbouring parts. Both the oefophagus and trachea indeed feemed to be covered with a thicker mucus than ufual; but his not having fwallowed any liquid during fifty-fix hours, may eafily account for this appearance. The left lobe of the lungs, and the edge of the great lobe of the liver, appeared to be inflamed; all the other vifcera were in their natural ftate.

It appears from the relation of this cafe, as well as from other recent ones, that the Ormfkirk powder does not poffefs any prophylactic

 power power with regard to this difeafe; neither is much ftrefs to be laid on the practice of dipping the patient in the fea, in the manner it is commonly ufed, to prevent this complaint: any effects it may produce feeming to be owing more to the impreffion of fear on the nervous fyftem, than to the common tonic power of cold bathing.

There feems reafon to fufpect that a dog, without having the hydrophobia upon him, is able to communicate that difeafe by a bite, as this man's dog had not exhibited any fymptoms of hydrophobia previous to its biting him; but it was killed too foon to afcertain this point with refpect to this dog. I am informed, however, from the beft authority, that the dog which bit Admiral Rowley's fon * two years ago, ate a plate of meat, and lapped a bafon of milk and water, after it had bit him; and fhewed no inclination to bite Sir James Cockburne's coachman, who tied a ftring round its neck, and led it to the coachhoufe, where it lived four days.

As this poor man remained well fo long, and had never experienced any uneafinefs in the

* See his cafe defcribed in the feventh volume of this Journal, page 90.

part part where he was bit, is there not reaſon to ſuſpect that an increaſed irritability of the nervous ſyſtem, from the ſtate his mind had been in for ſome time, might act as an occaſional cauſe of the diſeaſe? If ſo, might not good effects have been expected from a continued courſe of cold bathing, aſſiſted by ſome of the more powerful tonic medicines, eſpecially as mercurial courſes have failed in preventing this diſorder?

As cold bathing appears to have been of ſervice in ſome caſes of locked jaw, might not it be adviſeable to give it a fair trial in hydrophobia *?

Celſus, with his uſual brevity, recommends (as well as other authors) cold bathing; and from his ſaying "*Unicum* tamen remedium "eſt," one might ſuppoſe it had been found ſucceſsful in ſome caſes; but his idea ſeems rather to have been to force the patient to ſwallow drink, as he adviſes his being puſhed under water in caſe he can ſwim, and concludes with ſaying, "Sic enim ſimul et ſitis et aquæ metus "tollitur †."

* This was done in one of the caſes related by Dr. Vaughan, but without any eſſential advantage. — See his Caſes and Obſervations on the Hydrophobia, page 35. Editor.

† Celſus de Medicina, Lib. v. cap. 27.

No new light is thrown upon the rabies canina by this caſe; the only concluſion we can draw from it is, that we are as yet equally ignorant of the nature, the prevention, and the cure of this dreadful diſeaſe.

*Richmond,*
April 8, 1787.

